# Single-Cell Protein Production as a Strategy to Reincorporate Food Waste and Agro By-Products Back into the Processing Chain

**DOI:** 10.3390/bioengineering9110623

**Published:** 2022-10-28

**Authors:** Norma Julieta Salazar-López, Gabriel A. Barco-Mendoza, B. Shain Zuñiga-Martínez, J. Abraham Domínguez-Avila, R. Maribel Robles-Sánchez, Monica A. Villegas Ochoa, Gustavo A. González-Aguilar

**Affiliations:** 1 Facultad de Medicina de Mexicali, Universidad Autónoma de Baja California, Dr. Humberto Torres Sanginés S/N, Centro Cívico, Mexicali 21000, Baja California, Mexico; 2 Instituto Tecnológico de Sonora, 5 de Febrero No. 818, Centro, Urb. No. 1, Ciudad Obregón 85000, Sonora, Mexico; 3 Centro de Investigación en Alimentación y Desarrollo A. C., Carretera Gustavo Enrique Astiazarán Rosas No. 46, Col. La Victoria, Hermosillo 83304, Sonora, Mexico; 4 CONACYT-Centro de Investigación en Alimentación y Desarrollo A. C., Carretera Gustavo Enrique Astiazarán Rosas No. 46, Col. La Victoria, Hermosillo 83304, Sonora, Mexico; 5 Departamento de Investigación y Posgrado en Alimentos, Universidad de Sonora, Blvd. Luis Encinas y Rosales, Col. Centro, Hermosillo 83000, Sonora, Mexico

**Keywords:** protein bioengineering, sustainability, circular economy, macronutrients, industrial processes

## Abstract

Food waste is a serious problem with negative environmental and economic consequences. Unused food (either as waste or by-products and referred to as food residues in the present work) is a source of carbohydrates, lipids, proteins, vitamins, minerals and bioactive compounds that could be used in an alternate or secondary life cycle to avoid discarding it. The present work reviews the potential use of food residues for the bioengineering of single-cell protein (SCP), addressing aspects of production, nutrition and safety, as well as the main challenges and perspectives. SCP is obtained from various microorganisms, including fungi, bacteria, yeasts and algae, in pure or mixed form. SCP generally contains a higher percentage of protein (30–80%) compared to soy (38.6%), fish (17.8%), meat (21.2%) and whole milk (3.28%). SCP is a source of essential amino acids, including methionine, threonine and lysine. The use of food residues as substrates for the production of SCP would reduce production costs (35–75%); however, optimization and industrial scaling are some of the main challenges to its sustainable production. The use food waste and agro by-products from the food industry could be a promising alternative to obtain protein according to a circular production scheme.

## 1. Introduction

The world population is projected to reach 9.7 billion people by 2050 [1], causing an increased need to generate a greater volume of food, which is unsustainable under current production systems. Regarding protein, it is known that the preferred source of most consumers is meat products, according to its high consumption. For example, during the 2019–2021 period, average per capita meat consumption in North America, Oceania, Europe and Latin America was 98.5, 75.6, 63.2 and 60.9 kg, respectively; slight variations for these figures are projected for 2031 [2].

Current per capita meat consumption exceeds the recommended daily intake of the World Cancer Research Fund (less than 500 g per week) [3]. High meat consumption has been associated with various adverse health effects, including cardiovascular diseases, type 2 diabetes mellitus, cancer and even an increased mortality risk [4]. The World Health Organization (WHO) indicated that each 50 g portion of processed meat consumed daily increases the risk of colorectal cancer by 18% [5]; thus, consuming alternate protein sources could be a way to prevent some chronic non-communicable diseases that reduce quality of life and are an economic burden on health systems.

Most consumers do not appear to be tending towards reducing their meat consumption despite the cognitive dissonance caused by meat consumption and animal protection [6]. However, there is at least some openness to the acceptance of other protein sources than meat [7], for which new commercially available alternatives are necessary to satisfy the projected demands.

Various alternative protein sources currently exist, of which edible insects, plant-based protein alternatives, cultured meat and single-cell protein (SCP) are among the most representative [8,9]. SCP is also called bioprotein, microbial protein or protein biomass; it is obtained from various microorganisms, including fungi, bacteria, yeasts and algae, in pure or mixed form [10].

The production of unicellular protein rose partly due to the shortage of food that occurred in the 1950s and 1960s and nowadays in some parts of the world, and SCP has been considered an alternative to solve this problem. It is important to consider that protein-producing microorganisms can also be a source of complex carbohydrates (soluble and insoluble fiber), lipids, minerals and vitamins [10], further supporting their nutritional relevance. The use of microorganisms to obtain protein for food purposes has some advantages over conventional sources, including (1) the rapid growth rate of microorganisms and, thus, rapid protein production, since bacteria and yeasts double their population in 5 to 15 min, while algae and molds do so in 2 to 4 h [10]; (2) lower space requirements than protein from plant and animal sources, which need large areas of land; (3) reduced water requirements compared to plant and animal protein production; (4) SCP production is independent of climate or seasons, but it does require growth medium under controlled conditions, such as temperature, oxygen, light and continuous mixing [11]; (5) SCP does not require the use of insecticides, herbicides, fungicides and fertilizers, among other potential contaminants, which reduces environmental pollution and soil wear.

The production of SCP can be obtained from different microorganisms. Fungi have been known to benefit mankind in a number of ways, such as the production of antibiotics, drugs, recombinant vaccines and alcohol, in addition to land reclamation and bio-control [12]; its use as a protein-producing organism follows these previous uses. Fungi occur in the form of yeasts and molds, with yeasts being the most used microorganisms to produce SCP. The most prominent and most widely used yeast species are *Saccharomyces cerevisiae* and *Kluyveromyces marxianus*. These two species have become a significant research interest, since numerous scientific studies support their use to ferment different substrates and produce SCP with specific sensorial and nutritional characteristics.

Food biomass requires numerous resources to produce (water, land, fertilizer, man-power and others), with significant economic costs. Utilizing biomass that would otherwise go unused, such as bruised but otherwise safe-to-eat fruits/vegetables, would prevent wasting the resources that were initially invested on their production. Storing, handling, processing and properly disposing of unused food is also costly, and thus, utilizing it for SCP production would also minimize these costs, further leading to economic benefit.

Regarding SCP production for human consumption, it must use a food-grade substrate; however, tons of wasted food are discarded annually, which can be considered as a good source of unused nutrients [13]. At least a fraction of this is innocuous and can therefore be suitable to produce SCP as part of a circular economy system, which could positively impact food security and even lead to economic gains even for producers and agro-industrial processors. Redirecting food that would otherwise remain unused as input for a protein-producing process can be used to face the continuously increasing demand for this macronutrient while also minimizing the amount of food waste. The present work reviews the potential use of food residues from agroindustry to produce SCP regarding production, nutrition, and safety aspects.

## 2. Production

SCP can be obtained from a wide number of microorganisms, but they are limited when it is intended used for human consumption. Selecting the most appropriate species is influenced by its yield, growth rate, substrate, and optimal growth conditions, such as temperature, pH and nutrient requirements (particularly carbohydrates, vitamins and minerals). The selected microorganism should also be non-pathogenic, contain a low percentage of nucleic acids and must be innocuous [14]. The choice is also highly influenced by its protein yield; for example, this value ranges between 60 and 70, 30 and 80 and 30 and 50% *w*/*w* for microalgae, bacteria and yeasts, respectively [11]. However, these percentages can vary according to the substrate used for each case under optimal growth conditions.

**Figure 1:**
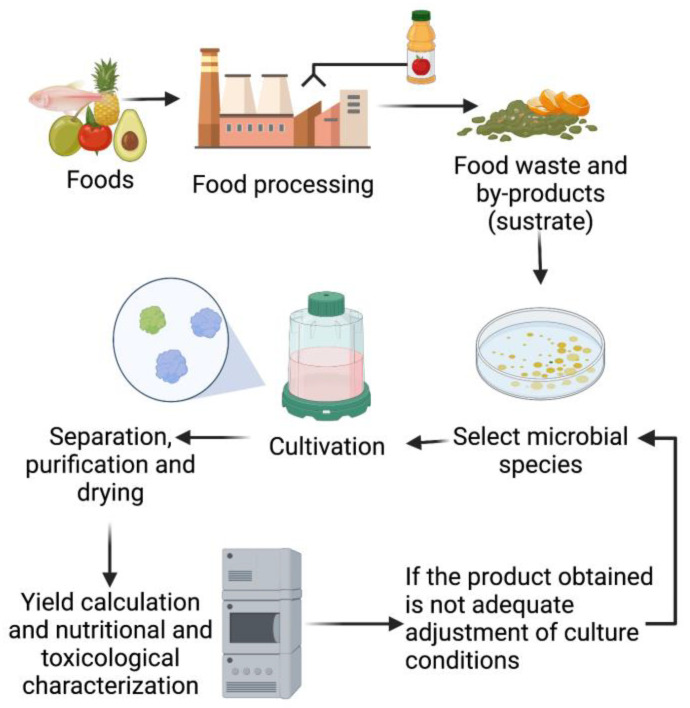
Screening and optimizing SCP production from food residues (food waste and agro by-products).

Regarding substrate, food residues are a source of carbohydrates, lipids, proteins, vitamins, minerals, and bioactive compounds [13,15]. Innocuous residues generated from the food industry could thus be considered as substrates for this process, reducing in consequence the amount of food waste. This approach can give added value to unused matter by allowing it to re-enter the food production chain as an important step in the practice of the so-called circular economy and can also contribute to global food security. The main SCP production process would reduce its production costs if the input material used (substrate) is of low monetary cost [10,16]. Therefore, the objective of SCP production focuses on the use of by-products from the food industry, which are naturally rich in different substrates that are normally used for growing microorganisms and whose cost can be nearly zero. The main process to generate SCP is shown in Figure 1, while previous investigations into using discarded food as substrate to produce SCP are summarized in Table 1.

It is important to mention that the substrate must contain nutrients (particularly carbon and nitrogen) in a form that is accessible to the microorganism, such as monosaccharides and disaccharides [10]. In this regard, it has been reported that a higher proportion of fermentable carbohydrates increases SCP yield; for example, *S. cerevisiae* cultivated in a potato-peel-based substrate with 82.32% carbohydrates showed higher yield compared to carrot pulp, banana peel and orange peel, whose carbohydrate contents were 61.86, 59.00 and 54.17%, respectively [17]. Additionally, it has been shown that regulating the carbon source and its conversion capacity can be achieved by including volatile fatty acids in the waste fermentation liquid, causing an increase in biomass production by photosynthetic bacteria. This is due to stimulating activity of metabolic pathways that can transform volatile fatty acids into tricarboxylic acids and/or acetyl-CoA, both critical pathways involved in the production of SCP [18].

The presence of antimicrobial compounds should also be considered since they can inhibit their growth and, thus, protein production. For example, a decrease in yield due to the presence of limonene, terpenes and camphene contained in citrus peels has been reported [17]. If the substrate contains significant amounts of compounds with similar antibacterial effects, it is necessary to apply pretreatments before it can be used. Autoclaving has been reported as a simple method for this purpose, since it can decrease the concentration of limonene in orange peel by 62%, which improves the growth of *S. cerevisiae* [19], but it varies according to the specific compounds.

Temperature and nitrogen sources are other important factors that must be considered during SCP production. The yeast *Kluyveromyces marxianus* (QPS in the European Union and GRAS in the United States) has a faster growth even at high temperatures (>40 °C) compared to *K. lactis* (GRAS), and most of its strains do not carry out alcoholic fermentation, which is desirable in SCP production where ethanol is undesired [20]. *K. marxianus* cultured on cheese whey lactose had increased protein content in response to increased fermentation temperatures, while its protein content could be further stimulated by other means. For example, adding ammonium salts induced a 45% (dw) protein increase, with a positive correlation between the concentration of ammonium sulfate (up to 2 g/L) and protein content, although it minimally reduced its biomass [21].

Adequately supplementing the culture medium with nitrogen is strictly necessary; if done at a 10:1 carbon: nitrogen ratio, it closely approximates what is generally found in microorganisms, although each particular species may require optimization [10]. In this regard, it has been reported that the addition of ammonium sulfate > ammonium nitrate > (sodium nitrate, corn steep or liquor-urea) to *S. cerevisiae* culture medium increases SCP yield when derived from industrial fermentation of food residues [17]. However, it is also important to control the source and concentration of nitrogen since high concentrations of ammonium ion (NH_4_^+^) negatively influence growth and tolerance to high temperatures of *K. marxianus*, which may require alternating organic nitrogen sources [22].

Once the microorganisms have reached a sufficient biomass, SCP must be isolated. These isolation processes consist of various extraction and purification steps that can increase time, energy use and cost of production, and may reduce sustainability. Once SCP has been isolated, it can be transformed into ingredients, a step whose complexity and cost depends on the purity or applications desired. To obtain the protein from the biomass, it is necessary to concentrate the producing organism and eliminate the aqueous medium in which it was contained through dehydration/drying. The harvest of microalgae, for example, can be carried out by coagulation/flocculation, filtering, and centrifugation, among other methods. Once the biomass is dry, and because its protein is contained inside the cells, it is necessary to disrupt their membranes and break the cells open. Cell disruption can be carried out through physical, chemical or biological methods, for example, pulsed electric fields, hydrodynamic cavitation or changes in pressure, enzymes or alkalis, among others [23,24]. After this step, the product consists of various-sized particles (depending on the lysis method used) with all their constituents mixed, including lipids, proteins, nucleic acids, etc.; protein purification then proceeds mainly by (ultra) centrifugation and filtration, followed by protein fractioning according to its solubility. Once fractionated, proteins are concentrated by dehydration, precipitation, ultrafiltration, spray drying and lyophilization, among other methods. Protein isolation can also be carried out according to their dispersibility, density or size (these processes have been covered in detail by other authors [23]). The processes are necessary but can be costly, thereby increasing the final cost of the products obtained.

Some authors report following similar strategies; for example, Böcker et al. [25] obtained protein from *Arthrospira platensis* microalgae by performing the following sequence of steps and consequent generation of effluents: they induced cell rupture with a microfluidizer, followed by centrifugation, precipitation by pH modification (isoelectric point), mixing, centrifugation and purified by tangential-flow diafiltration [25]. These processes are energy-intensive, which is consistent with those reported by Smetana et al. [26], who mention that the production of a mycoprotein-based meat substitute is associated with a high energy demand. They also mention that the mycoprotein-based product has a lower environmental impact than laboratory-grown meat but shows superior impact to chicken meat or dairy- and gluten-based meat substitutes [26], which is an argument in favor of further research to optimize SCP production processes.

**Table 1:** Previous research where food waste was used as substrate to produce SCP.

Microorganism	Substrate	Biomass Yield	Protein Content (%)	Reference
Yeasts				
*Saccharomyces cerevisiae*	Fish, pineapple, banana, apple, and citrus peels	NR	40.2%	[27]
*Saccharomyces cerevisiae*	Food wastes (banana peel, citrus peel, carrot pomace and potato peel)	Up to 12 g/100 g of waste	47.7%	[17]
*Saccharomyces cerevisiae*	Candy production effluent and digestate	0.25 g/L per day	28.0%	[11]
*Saccharomyces cerevisiae*	Vinasse	73.2 g/L per day	53.3%	[28]
*Yarrowia lipolytica*	Unspecified food waste	8.4 ± 0.7 g/L	38.8%	[29]
*Candida utilis*	Orange peel residues	15.7 g/L	6.2%	[30]
*Candida tropicalis*	Soy molasses	8.4 g/L	53.1%	[31]
*Kluyveromyces marxianus*	Food industry waste mixture	NR	33.7%	[32]
*Hanseniaspora guilliermondii* and *Issatchenkia orientalis*	Wasted date molasses	Up to 70 g/100 g of waste	54.3%	[33]
Fungi				
*Aspergillus niger*	Orange peels	NR	29.7%	[34]
*Geotrichum candidum (fungi) and Candida utilis (yeast)*	Yellow wine lees and rice soaking waste *fungi:yeast* (1:1)	4.91 g/g of waste	68.5%	[35]
*Fusarium moniliforme (fungi)* and *Saccharomyces cerevisiae (yeast)*	Sweet potato residue	13.9 g/L	65.8%	[36]
Algae				
*Haematococcus pluvialis*	Synthetic brewery wastewater	27 × 10^5^ cells/mL	64.9%	[37]
*Chlorella* sp.	Food processing wastes (tofu)	42.5 × 10^6^ cells/mL	52.3%	[38]
*Chlorella vulgaris* (microalgae) and *Yarrowia lipolytica* (yeast) and mix culture	Liquid digestate of dairy wastewater	*C. vulgaris* 0.13 g/L per day; *Y. lipolytica* 0.1 g/L per day; Mix 0.2 g/L per day	*C. vulgaris* 21.8% *Y. lipolytica* 25.7% Mix 31.1%	[39]
Bacteria				
*Brevibacterium lactofermentum* and *Candida utilis* (yeast)	Beet pulp	53 g/100 g of waste	54.5%	[40]
*Methylomonas and Methylophilus*	Sewage sludge and discarded effluent	11.54 g/g-NH_4_^+^	41%	[41]
*Rhodopseudomonas faecalis*	Sugar industry wastewater	2.5 g/L per day	51.5%	[42]

NR: Values not reported.

## 3. Nutrient Content

As previously mentioned, the quantity and quality of SCP depends on the microorganism used and its culture conditions; for example, protein yield of microalgae can vary widely from 30 to 80% [43]. SCP generally contains a higher percentage of protein than soy (38.6%), fish (17.8%), meat (21.2%) and whole milk (3.28%) [44]. Table 2 shows a comparison of protein and amino acid content between conventional foods and SCP. The SCP from *H. pluvialis* and *S. cerevisiae* is a source of threonine (up to 7.41%) and tryptophan (up to 14.22%), both of which are considered limiting amino acids in milk and meat, respectively [28,37,45]. SCP is a source of the essential amino acids methionine, threonine and lysine, with the latter is deficient in cereals, suggesting they could be mixed to obtain a more nutritive food.

It has been reported that SCP protein could be higher than that of other protein sources, which can potentially exert some benefits to an organism which consumes it. For example, the addition of a methanotroph (*Methylococcus capsulatus*, Bath) to the diet of Jian carp (*Cyprinus carpio* var. Jian) significantly improved mean final weight, rate of weight gain, specific growth rate and serum antioxidant capacity while significantly decreasing malondialdehyde production compared to fish fed soybean meal [46]. This is consistent with other reports, where an increased growth of farmed shrimp was attributed to the substitution of more than 20% of fishmeal by biofloc meal (mixture of microorganisms) [47].

## 4. Safety

SCP may contain substances that may be harmful to human health, such as toxins, nucleic acids (RNA), allergens, pathogens, pesticides and heavy metals, among others [48]. The content of nucleic acids (RNA) in SCP can represent a problem for human health, since high purine consumption can increase the concentration of uric acid (hyperuricemia), which is a precursor to diseases like gout or kidney stones [16]. A preventive strategy regarding nucleic acids is selecting an appropriate microorganism which normally contains low concentrations, for example, algae over fungi and bacteria. Other options have also been evaluated to reduce it, including enzyme (ribonucleases) or thermal treatments or adding salts, acids or hydroxides [16,17]. In this regard, various treatments to decrease nucleic acid concentration of *S. cerevisiae* produced when cultured on potato peels was reported, wherein a heat shock treatment (70 °C for 80 s), a base (NaOH, 1 N), acid (HCl, 2%) and salt (NaCl, 2%) reduced it by 43%, 36%, 20% and 17%, respectively [17].

The presence of toxins produced by microorganisms also requires attention. This can be prevented with careful selection of species, substrate, and medium conditions, for example, preferring algae over fungi since some fungal species can produce mycotoxins with allergenic or carcinogenic potential. Some microorganisms are also capable of producing toxins (exotoxins and endotoxins), including mycotoxins, ochratoxins and aflatoxins, among others, whose effects on human health can be significant. In particular, around 50 species of *Fusarium* are producers of mycotoxins (fumonisins), which can induce damage to the central nervous system [49], thus restrengthening the argument that selecting the most adequate microorganism is of utmost importance.

Allergic or similar events have also been reported in humans due to the consumption of microorganism-based foods or supplements. For example, the consumption of Spirulina (*Arthrospira platensis*) tablets induced an anaphylactic event on an adolescent, where the presence of phycocyanin was identified as responsible [50]. A case was also documented where a boy consumed *Chlorella* tablets for 3 months, which lead to acute tubulointerstitial nephritis [51]. Thus, allergies or sensitivities to the microorganisms must also be considered.

Other specific allergens, contaminants or harmful substances from the substrate could be reduced with a careful screening process, particularly if it comes from food waste or from another industry where careful treatment is not considered. Therefore, single substrates such as glucose, dextrose or sucrose may also be considered as possible supplements for the process. These would minimize harmful substances that could be contained in the substrate, thereby promoting a high level of safety for products meant for human or animal consumption.

Treatments to eliminate toxic substances or potential contaminants should ideally be performed minimally if previous precautions are taken into consideration, such as carefully selecting the microorganism and utilizing innocuous substrates. Otherwise, additional processing steps could reduce yield and/or increase cost.

## 5. Applications and Challenges of Single-Cell Protein

SCP obtained from the fermentation of food residues has been proposed as a suitable protein source that could be used in various formulations (such as protein supplements) for human and animal nutrition. This section highlights some of the possible applications that have been considered by various authors.

Hashem et al. [33] used date molasses (a by-product derived from their manufacture), which are able to support the growth of a large yeast biomass and, therefore, SCP. The authors report a high concentration of reducing sugars (73.12%), which made it suitable to incorporate into yeast growth medium, where it improved fermentation parameters. Because these date by-products could be successfully transformed into SCP, it was reported that they could be used as a substrate that supplies carbon and minerals for its production. They indicate that the SCP could be used industrially as a supplement for animal feed; however, this was not specifically tested in an animal model to support this application, which highlights the need for further experimentation. Further investigations are also needed to suggest its possible use as ingredient in human foods, in addition to approval by relevant authorities (such as the FDA in the USA), as well as to determine its economic feasibility in comparison with other protein sources of animal or plant origin.

The industrial processing of coffee generates large amounts of wastewater, which has been used as a biotechnological alternative to obtain a rich nutrient extract containing high amounts of sugars, proteins, and salts. Pillaca-Pullo et al. [52] observed that a yeast (Candida sorboxylosa) was able to successfully assimilate glucose, mannose and fructose (reducing sugars) from this substrate, resulting in high SCP yields of 37.4% to 39%, which could be used as an economical alternative protein source for animal feed supplements. Similarly, wastewater from potato peels (supplemented with 5% glycerol) was used as a carbon source by Kurcz et al. [53], who observed that Candida utilis was able to transform it into SCP, with a yield of 30 g dw/L and an efficiency in protein biosynthesis of 12.2 g/L. They argue that, because this yeast has been approved by the FDA as safe for consumption, its biomass and metabolites can be used both in the food industry as well as in the production of animal feed. According to this evidence, wastewater from different industries could be transformed into SCP; however, the applications proposed of using it as animal feed or supplement were not analyzed by the authors in any animal model, and thus, further experiments are still required to verify this potential application.

The production of SCP from food waste is one of the most promising approaches, since it is an economical source of biomass, in addition to being environmentally friendly. Khan et al. [17] fermented vegetable residues (banana peel, citric residues, carrot pomace and potato peel) for SCP production, highlighting the higher yield of potato peel. The authors then incorporated their obtained SCP into wheat flour-based breads, where they observed that adding up to 4% SCP did not negatively affect its organoleptic properties. This result indicates the viability of using these by-products to produce SCP that could then be used to supplement other edible products. Because they also reported that the SCP obtained was rich in essential amino acids, it could be used to enrich wheat flour-based products and improve its nutritional properties. In addition to the reported sensory properties, further studies are still required to determine its possible health bioactivities in vitro or in animal models before considering human nutrition.

Carranza-Méndez et al. [30] used orange peel as substrate for Candida utilis to produce SCP by submerged fermentation. The authors highlight it as an excellent source of carbohydrates and minerals, particularly calcium and potassium. They argue that the SCP obtained could be used in the food industry; however, they mainly focused on the development of the bioprocess to obtain it, while its application in an edible product was not specifically studied, suggesting the need for further experimentation.

The use of wastewater and various food residues/wastes as low-cost substrates to produce SCP can reduce their environmental impact. Although the evidence is promising, this is a relatively new area of study that has not yet been fully explored. Many potential applications have been proposed by authors who have produced SCP from various sources, but most of them have not been thoroughly analyzed. Their impact on the physicochemical and organoleptic properties of a product in which it is added, or its effects on health have not been determined in detail. There are also few in vitro or in vivo studies of its possible bioactivities, since most authors are currently focusing on developing and optimizing the process used to obtain SCP. Therefore, various complementary experiments within this broad field are needed to fully explore its real-world applications, which remain as challenges for the upcoming years/decades. It has been reported that the demand for protein will significantly increase in the next decades. These analyses into the uses and bioactivities of SCP must be performed since the need for alternative protein sources and innovation in this regard is constantly increasing. This requires concerted efforts by various actors, particularly by different businesses and investors, whose economic and/or technological capacity can be focused on solving the different challenges that this developing industry is facing [9]. Effective and rapid regulation by government agencies is also necessary in order to promote nutritious and safe protein alternatives (SCP in this case, whose composition is suitable to be included in foods for human consumption). However, toxicological analyses, including for heavy metals, minerals, and RNA, among others, have to be performed in order to avoid any possible negative health effects to consumers.

Another major challenge is how SCP can be incorporated into the everyday diet. There are currently various SCP-based products on the market [16]; however, they remain a niche product that is not widely available or consumed. This can be partially solved by developing and offering the consumer options that are made from or contain SCP, offering SCP-based dishes in restaurants to popularize them with a wider section of the market or producing functional foods with increased nutritional properties [54]. In this regard, the addition of Arthrospira platensis (4%) to a white chocolate formulation increased its protein, amino acid, lipid and mineral contents by 23.1%, 45%, 10.3% and 13.5%, respectively, without modifying its sensory acceptance [55]. This suggests that SCP could be incorporated into a wide number of foods with the purpose of increasing their nutritional value and potentially inducing health benefits due its content of essential amino acids, peptides and minerals, among others.

Consumer acceptance also requires significant effort, perhaps even higher than that reported for cultured meat and plant-based meat alternatives [9], in order to maximize potential market adoption. Consumer acceptance may be promoted by highlighting the potential health benefits of SCP. In this regard, peptides with antioxidant capacity and inhibitory potential against angiotensin-converting enzyme (ACE) have been obtained from Arthrospira plantensis [56], a bioactivity that can contribute to maintaining healthy blood pressure. Similar findings from other sources have also been described, such as an ACE-inhibiting peptide obtained from a K. marxianus protein hydrolyzate [57]. Asmaz and Seyidoglu [58] have shown that consuming Spirulina platensis (Arthrospira platensis) for 45 days exerts intestinal health benefits on Wistar rats, according to increased expression of proliferating cell nuclear antigen (PCNA) and proliferation index [58]. Others report that an astaxanthin-rich SCP was obtained from Haematococcus pluvialis that was exposed to red LED light which exerted a significant antioxidant potential (according to DPPH radical scavenging activity) [37].

Although there are numerous reports about the nutritional value and health-promoting effects of SCP, additional research is required, particularly to verify the properties of products that come from large-scale industrial sources, since most data are derived from samples that were obtained at the laboratory level.

Finally, the efficiency of C and N utilization from food waste should be clearly analyzed in a standardized manner, in order to compare between various substrates and against traditional sources of these elements. Such data would also make it possible to determine the economic benefits of re-introducing this biomass more precisely back into the food processing chain, in order to make it more attractive for investors, producers and consumers while also providing concrete data to policy makers about the economic gains from this practice, which would further advance this growing field.

## 6. Conclusions

SCP obtained from food residues (including food waste and by-products) can have a higher protein content compared to conventional foods like meat, milk and fish and can contain essential amino acids. The inclusion of SCP in foods has shown sensory acceptability, even superior to laboratory-grown meat and plant-based protein alternatives. Some constituents of SCP have shown potential health benefits, including antioxidant potential, inhibitory potential against angiotensin-converting enzyme and intestinal health benefits. SCP is a promising alternative protein source, but there are still some challenges in its production, such as industrial scaling with profitable yields, optimization of isolation processes, purification and elimination of toxic substances, in addition to the additional experiments required to validate its use in animal and/or human nutrition (or other purposes). The development of alternatives and innovation regarding new protein sources still requires encouragement by various actors, including businesses, investors, academics and governments, whose economic, scientific, technological or legislative support can induce significant changes. The technical challenges listed throughout the manuscript require cooperation from various research groups in order to be adequately addressed, all with continued funding by governments and private entities. As a relatively new field, SCP production lacks gold standard methods that can be universally used, but as research is focused on the subject, its aim should be placed on standardizing them to obtain increasingly faster and more substantial progress. Growth in this field is in the interest of all modern societies as food security becomes an increasingly vital issue. The bioengineering of food waste and agro by-products from the food industry could be a promising alternative to obtain protein within a circular production scheme, which can exert various nutritional, environmental, and economic benefits.

## Figures and Tables

**Table 2:** Comparison of the nutritional composition of conventional protein sources and SCP.

Components	Meat (Beef) [44,45]	Milk (Cow, Whole) [44,45]	Fish (Carp, Raw) [44,45]	*Rhodopseudomonas faecalis* (Bacterium) [42]	*Candida utilis* (Yeast) and *Brevibacterium lactofermentum* (*Corynebacterium*) [40]	*Saccharomyces cerevisiae* (Yeast) [27]	*Saccharomyces cerevisiae* (Yeast) [17]	*Saccharomyces cerevisiae* (Yeast) [28]	*Haematococcus pluvialis* (Microalgae) [37]
Lipid %	3.68	3.2	5.6	NR	0.10	14.4	2.3	NR	NR
SFAs (%)	1.65	1.86	1.08	NR	NR	24.68	NR	NR	NR
MUFAs (%)	1.22	0.69	2.33	NR	NR	47.02	NR	NR	NR
PUFAs (%)	0.22	0.11	1.43	NR	NR	26.39	NR	NR	NR
Ash %	1.02	NR	1.46	NR	11.5	1.08	7.85	NR	NR
Fiber %	0	0	0	NR	14.2	NR	3.38	NR	NR
Carbohydrate (%)	0.23	4.67	0	NR	NR	NR	34.88	NR	NR
Protein %	21.2	3.28	17.8	51.5	54.5	NR	47.7	53.31	64.93
Essential amino acids (%)									
Isoleucine	2.41	0.12045	2.71	3.7	3.45	NR	2.12	1.27	2.58
Leucine	4.06	0.2365	4.35	7.6	4.13	NR	4.35	2.28	10.87
Lysine	4.45	0.1364	5.16	5.6	25.00	NR	3.14	0.28	0.47–11.05
Methionine	1.35	0.0473	1.62	0.5	1.86	NR	1.12	NR	0.54
Phenylalanine	2.20	0.13145	2.22	4.1	1.65	NR	2.69	1.84	2.07–3.17
Threonine	2.29	0.08415	2.59	0.3	3.93	NR	2.49	3.03	1.59–7.41
Tryptophan	-	-	-	3.8	NR	NR	NR	14.22	ND
Valine	2.50	0.14025	3.46	5.5	2.81	NR	3.84	3.49	ND
Histidine	1.70	0.0649	2.00	1.9	3.12	NR	0.79	3.3	0.34–1.84
Non-essential amino acids (%)									
Cysteine	0.64	NR	0.66	1.0	NR	NR	0.19	NR	0.55–1.19
Tyrosine	1.80	0.12	2.07	2.5	2.49	NR	0.52	7.24	1.11–6.91
Arginine	3.16	0.05	3.21	1.1	3.35	NR	3.21	1.76	5.55–21.44
Alanine	2.92	0.08	3.39	6.6	2.82	NR	1.84	2.17	1.15–12.68
Aspartic acid	4.50	0.13	5.86	4.7	4.87	NR	3.9	6.27	7.24–18.71
Glutamic acid	7.65	0.35	7.99	3.7	12.00	NR	NR	7.83	0.85–5.62
Glycine	2.43	0.04	2.73	6.1	3.87	NR	1.52	5.6	9.38–28.12
Proline	1.89	0.15	2.08	5.4	2.74	NR	0.39	32.07	6.45–9.96
Serine	2.02	0.10	2.45	3.7	1.24	NR	1.44	9.31	2.72–7.7
Asparagine	NR	NR	NR	NR	NR	NR	1.39	NR	0.68–6.67
Glutamine	NR	NR	NR	4.3	NR	NR	2.04	NR	1.53–6.83
Limiting amino acid	Tryptophan	Threonine	Isoleucine						

NR: Values not shown were not reported in the respective experiment. ND: not detected.

## Data Availability

Not applicable.
